# Patient safety indicators for virtual consultations in primary care: A systematic review protocol

**DOI:** 10.1371/journal.pone.0313639

**Published:** 2025-01-09

**Authors:** Tetiana Lunova, Ulrik Bak Kirk, Geva Greenfield, Andrée Rochfort, Ara Darzi, Ana Luisa Neves

**Affiliations:** 1 NWL Patient Safety Research Collaboration, Institute of Global Health Innovation, Imperial College London, London, United Kingdom; 2 Department of Public Health, Aarhus University, Aarhus, Denmark; 3 Research Unit for General Practice, Aarhus, Denmark; 4 Department of Primary Care and Public Health, Imperial College London, London, United Kingdom; 5 Irish College of General Practitioners, Dublin, Ireland; 6 School of Medicine, University College Dublin, Dublin, Ireland; University of South Australia, AUSTRALIA

## Abstract

**Background:**

Virtual consultations are being increasingly incorporated into routine primary care, as they offer better time and geographical flexibility for patients while also being cost-effective for both patients and service providers. At the same time, concerns have been raised about the extent to which virtual care is safe for patients. As of now, there is no validated methodology for evaluating the safety nuances and implications of virtual care. This study aims to identify patient safety indicators that could be used to evaluate the safety of virtual consultations in primary care.

**Methods:**

A literature search will be performed in Ovid MEDLINE/PubMed, Embase, and Cochrane Library for relevant articles published over the last 10 years (2014–2024). The systematic review will include randomized and non-randomized controlled trials and observational studies with adult populations that compare synchronous patient-provider virtual consultations (telephone or video) or multicomponent interventions involving synchronous remote consultations with face-to-face consultations. The outcome of interest will be patient safety indicators extracted from the studies. The quality of randomized controlled trials will be assessed with the Cochrane Risk of Bias Tool, and the Newcastle-Ottawa Scale will be used to analyze risk of bias in observational studies.

**Discussion:**

Considering the growing adoption of virtual medical care worldwide, a robust and comprehensive evaluation of its safety and quality is now a system-wide priority. Therefore, one of the primary strengths of this proposed systematic review is its focus on a topic of great importance and timeliness, specifically addressing the existing knowledge gap in this area. By publishing this protocol, we demonstrate the transparency and reliability of our research strategy and aim to minimize the risk of selection bias. Potential limitations include the heterogeneity of measures and outcomes, as well as a lower-than-expected number of studies in subgroup analyses, which may negatively influence the statistical significance in data synthesis.

**Trial registration:**

**PROSPERO registration number:** CRD42023464878.

## Introduction

Patient safety, defined as “the prevention of unintended and avoidable harm caused to patients during the provision of medical services,” is a fundamental component of robust and high-quality healthcare [1–3]. Prioritizing patient safety not only leads to better patient outcomes but also enhances cost-efficiency by reducing unnecessary additional treatments resulting from medical errors. To effectively capture existing patient safety issues and inform healthcare providers in their decision-making towards quality improvement, it is critical to have a robust strategy in place. According to existing evidence, Patient Safety Indicators (PSIs) have emerged as one of the most effective methods of safety evaluation in healthcare [4–6].

PSIs are a set of measures that evaluate the avoidable harm to patients resulting from the use of healthcare services and provide a perspective on the quality and safety of these services [5]. They help healthcare providers determine problem areas by tracking avoidable patient safety incidents and identifying patients at increased risk of harm from using the service [4]. One of the main benefits of PSIs is their quantitative surveillance, which enables healthcare providers to calculate proportions or rates of patient safety events in a population sample (using rate-based PSIs) or identify sporadic undesirable events (using sentinel indicators) [4, 7]. PSIs can also be useful in evaluating the safety impact of organizational changes. Unlike a qualitative, case-based approach to patient safety, the quantitative aspect of PSIs allows for more precise reporting and safety analysis [7]. PSIs can be designed to detect events related to preventable patient safety risks and, consequently, prompt further analysis, investigation, and priority setting. They may also indicate preventable clinical adverse outcomes and reflect clinical or organizational aspects (staff, equipment, treatment, investigation, etc.) that relate to patient security [8].

Several sets of indicators have been successfully applied in evaluating patient safety across different levels of healthcare in Europe and the US [6]. One of the most widely known lists of indicators has been developed by the Agency for Healthcare Research and Quality (AHRQ) [4, 5]. The AHRQ PSIs focus on potential in-hospital complications and adverse events following operations, procedures, and childbirth [9]. The AHRQ list includes 26 indicators, such as “Pressure Ulcer Rate,” “Birth Trauma Rate–Injury to Neonate,” and “Death Rate in Low-Mortality Diagnosis-Related Groups (DRGs).” Similarly, the ESQH-office for Quality Indicators worked on the PSI set for the European Union health system, covering surgical complications, infection control, obstetrics, falls, and medication errors [7].

However, the above-mentioned sets of indicators are mostly tailored to evaluate safety events in hospital care and cannot be reliably used for safety evaluation at other levels, such as primary care. Patient safety incidents and quality improvement in primary care have been investigated in a significant body of evidence, including a study by Engels et al., who developed a framework and quality indicators for general practice management [10–13]. Meanwhile, there is no validated set of patient safety indicators that could capture the nuances of virtual consultations.

Virtual consultations are being increasingly incorporated into routine primary care as they offer better time and geographical flexibility for patients while also being cost-effective for service providers [14, 15]. At the same time, concerns have been raised about how safe virtual care is for patients, especially for those with cognitive or hearing impairments, frail patients, people with language barriers, or low digital literacy [16]. Considering that virtual consultations have already become an indispensable part of today’s healthcare, a robust method adapted to capture associated safety challenges is needed.

This systematic review will explore patient safety indicators that could be used to evaluate the safety of virtual consultations in primary care, delivered via telephone or video either alone or as a part of a multicomponent intervention (e.g., virtual consultation+telemonitoring).

### Research aims

The objective of this study is to systematically review, extract, and summarize relevant patient safety indicators that could be used in the context of virtual consultations in primary care, encompassing the variety of service delivery models currently in use (*i*.*e*., telephone, video consultation). As a secondary aim, we will develop a thematic analysis to map identified PSIs into a comprehensive framework for patient safety in primary care.

## Materials and methods

The protocol has been developed in accordance with PRISMA-P guideline by Moher et al. (S1 File) [17].

### Search strategy

A literature search will be performed in Ovid MEDLINE/PubMed, Embase, and Cochrane Library for relevant articles published over the last 10 years (2014–2024). This time frame was deemed relevant to capture advancements in virtual medical care which have taken place over the last decade. We will also identify eligible studies by checking the reference lists of included articles to capture any studies that may have been missed in the data base searches and explore grey literature sources, such as gov.uk data on Medicine safety, European Frameworks for patient safety in primary care (i.e., LINNEAUS EURO-PC) [18, 19]. Search strings will combine free terms and controlled vocabulary, such as “virtual consultation”, “remote consultation”, “online consultation”, “patient safety”, “patient harm”, “medical errors”, etc. An initial literature search of Ovid MEDLINE database was performed to determine the feasibility of the review, look at the amount and quality of available data and refine the search strategy. The free terms and controlled vocabulary were identified and agreed through multiple discussions with the academic liaison librarian of Imperial College London as well as utilizing the guidance on systematic reviews from Imperial College London. An example of a search strategy is presented in a supplementary information file (S2 File).

### Study selection

The systematic review will include experimental studies (randomized and non-randomized control trials) and observational studies (cohort, case-control, and cross-sectional studies) with the adult population. Descriptive studies and studies that involve children and adolescent population will not be considered. We will include studies that evaluate 1) synchronous patient-provider virtual consultations delivered via telephone or videoconference by primary health care professionals, and 2) multicomponent interventions involving synchronous remote consultations. Any other digital health interventions that do not include virtual consultations with medical professionals (i.e., conversational agents) will not be considered. We will be looking for studies that compare remote consultations with face-to-face synchronous consultations in the primary care setting. Consultations delivered by non–health care professionals or specialist clinicians; or consultations delivered in retail clinics or via direct-to-consumer models will not be included. This review will focus on the most prevalent conditions managed in primary care according to NHS data, including, but not limited to, arterial hypertension, COPD, depression, diabetes mellitus, and others [20]. Studies investigating virtual care for acute and emergency conditions, oral health issues, end of life and palliative care will be excluded (Table 1).

**Table 1:** Study inclusion and exclusion criteria.

	Inclusion criteria	Exclusion criteria
Population	Adult patients.	Children and adolescents (under 18 years old).
Intervention	Synchronous patient-provider virtual consultations delivered via telephone or videoconference by primary care health care professionals, either alone or part of a multicomponent intervention. Prevalent diseases managed in primary care will be studied.	Digital health interventions that do not involve remote consultations. Consultations delivered by non–health care professionals or hospital-based health professionals; or consultations delivered in retail clinics or via direct-to-consumer models will not be included. Combined consultations that include face-to-face consultation as one of the components. Studies looking into dental care, end of life and palliative care management will not be considered.
Comparator	Consultations delivered face-to-face.	-
Outcome	Patient safety indicators extracted from the studies.	Any other measures that cannot be considered as patient safety indicators.
*Other criteria*		
Study type	Experimental studies (randomized and non-randomized control trials) and observational studies) will be eligible for inclusion.	Descriptive studies, systematic reviews and meta-analyses , editorials, conference proceedings, letters.

### Screening and data extraction

Deduplication and literature screening will be performed in Covidence software by two independent reviewers (TL and KHH) and reported using PRISMA flow diagram [21]. A preliminary assessment will involve screening of study titles and abstracts and selecting the potentially eligible studies for the full-text review according to pre-established criteria. Full-text screening will be performed by the same reviewers, and all disagreements will be resolved through discussion or by involving a third investigator. Cohen’s kappa will be used to measure interrater reliability in each screening phase [22].

Data extraction will be performed by two independent investigators into a structured form. Information collected from the studies will include study title, name of the first author, year of publication, sample size, disease studied, intervention and comparator measures, participants’ and setting characteristics, patient safety indicators (PSIs). The data extraction table will be piloted before use to identify any gaps in the data extraction strategy. The PSIs that emerge the same across different articles will be aggregated, with a number of reporting articles cited. The PSIs will be grouped by the condition studied and reported in the data extraction table. Any missing data will be confirmed via correspondence with authors. In case any critical data is missing, and no response is received from the authors, the study will be excluded. Two investigators will review the data extraction form to ensure consistency, and all disagreements will be resolved through discussion.

### Narrative synthesis and subgroup analysis

It is anticipated that conducting a meta-analysis will not be feasible due to the expected heterogeneity of findings. Instead, a narrative synthesis will encompass all included studies to outline main study characteristics and highlight observed findings. The narrative synthesis will provide summaries of the studies by major disease category. Alongside this synthesis, a table summarizing extracted patient safety indicators will be provided, detailing study descriptives such as population demographics, study design, diseases studied, and study locations/settings. Subgroup analyses are planned based on the modality of remote consultation (telephone vs. video), with potential additional subgrouping by specific diseases, geographical locations, settings, and relevant health system contexts. For studies exhibiting a high or unclear risk of bias (defined as 50% or more of quality assessment outcomes), a narrative description of the bias risk will be included.

### Quality assessment

The quality of randomized controlled trials will be evaluated at the outcome level using the Cochrane Risk of Bias Tool, covering domains including randomization process, intervention assignment and adherence, missing outcome data, outcome measurement, and selection bias in reporting results. Non-randomized studies (e.g., case-control, cohort) will be assessed using the Newcastle-Ottawa Scale, focusing on criteria related to study selection, exposure assessment, and outcome evaluation [23, 24].

Two independent reviewers (TL and KHH) will independently score selected studies, resolving any discrepancies through discussion or involving a third reviewer if necessary. A comprehensive risk of bias table accompanied by a narrative statement will summarize bias assessments across individual studies.

### Strength of evidence

The strength of evidence will be evaluated using the ’Grading of Recommendations Assessment, Development, and Evaluation’ (GRADE) criteria [25].

### Patient and public involvement

A discussion with the members of the North-West London Patient Safety Research Collaboration Research Partners Group was held to gain patient and public perspectives on the relevance and benefit of this study, as well as to determine the strategy for future work on the project. Patient partners will also be involved in interpreting the results and disseminating the findings of this systematic review and subsequent focus group study. More precisely, patients and the public will be engaged in translating the research into lay summaries for distribution and ensuring the applicability of the findings for wider audiences. At the end stages of the project, an open roundtable will be held to circulate the results and strengthen the relationships between our research team and the community.

In addition to patient and public involvement, an online webinar with leading research and policy professionals in the field will be held to discuss the current state of patient safety in primary care from the digital health angle. This webinar will explore expert ideas on using patient safety indicators in virtual consultations, gather public opinions on the applicability and feasibility of PSIs, and foster discussions around the practical aspects of PSI implementation from the providers’ point of view.

This review will lay the groundwork for our further research projects on the subject, which will involve patient partners in co-developing and finalizing a set of indicators and a safety framework.

### Ethics and dissemination

This review will analyze studies with non-identifiable information and, therefore, does not require ethical approval. Study findings will be disseminated through preprints, open-access publications in peer-reviewed journals, conference presentations, and policy documents.

## Discussion

### Research contribution and policy implications

Considering the growing adoption of virtual medical care worldwide, a robust and comprehensive evaluation of its safety and quality is now a system-wide priority. Current data shows that the use of virtual care in 2023 is 38 times higher than before the pandemic, and it continues to grow even though the COVID-19 emergency is over [26]. Therefore, one of the primary strengths of the proposed systematic review is the importance and timeliness of the selected topic. Developing a set of patient safety indicators (PSIs) for virtual consultations will make safety evaluations more transparent, robust, and reliable, while allowing for more precise reporting and safety analysis. Having a set of PSIs in place will reinforce documenting and benchmarking the quality and safety of virtual care across organizations. Findings from this review will enable the tracking of safety incidents in certain population groups or in patients with specific conditions or symptom patterns, thereby influencing guidance on when a virtual consultation is safe and should be offered.

### Strengths and limitations of the study design

In this study, we utilize a clear and transparent approach to synthesizing literature on the subject. This protocol sets clear inclusion and exclusion criteria and coherently describes what types of studies will be selected and how screening and data extraction will be performed. In cooperation with a specialist librarian, we developed a thorough search strategy to ensure all relevant studies are captured. Well-recognized quality assessment tools will be used to provide quality assurance of the included studies, while adherence to PRISMA-P guidelines will guarantee the clarity of reporting. By publishing this protocol, we demonstrate the transparency and reliability of our research strategy and act to minimize the risk of selection bias. Among the strengths of this study are the novelty and research value it can add to existing knowledge on the subject. Since there is a significant gap in evidence regarding patient safety evaluation of virtual consultations in primary care, this systematic review can make a pivotal contribution to current research.

Potential limitations include the heterogeneity of measures and outcomes evaluated. Another limitation that could arise in this review is a lower-than-expected number of studies in subgroup analyses, which may negatively influence the data synthesis.

### Amendments

Any amendments to this protocol will be documented with reference to saved searches and analysis methods, which will be recorded in bibliographic databases and Excel templates for data collection and synthesis.

## Supporting information

S1 File Checklist of the Preferred Reporting Items for Systematic Reviews and Meta-Analyses Protocols (PRISMA-P). (DOCX)

S2 File An example of a search strategy for Ovid MEDLINE. (DOCX)
