# Supplementary material for: Patient safety indicators for virtual consultations in primary care: A systematic review protocol
Source: PLoS One. 2025 Jan 9;20(1):e0313639. doi: 10.1371/journal.pone.0313639 (PMC11717205; doi:10.1371/journal.pone.0313639)
Supplement: S1 File — (DOCX) [file pone.0313639.s001.docx]

**Literature search strategy**

**Database:**
Ovid MEDLINE(R) ALL <1946 to April 10, 2024>

| **#** | **Query** | **Results from 11 Apr 2024** |
| --- | --- | --- |
| 1 | Telemedicine/ or Remote Consultation/ | 44,028 |
| 2 | (online consultation* or remote consultation* or virtual consultation* or video consultation* or virtual care or remote care or telemedicine or tele medicine or telecare or tele care or teleconsultation* or tele consultat* or telehealth or tele health or virtual visit*).mp. [mp=title, book title, abstract, original title, name of substance word, subject heading word, floating sub-heading word, keyword heading word, organism supplementary concept word, protocol supplementary concept word, rare disease supplementary concept word, unique identifier, synonyms, population supplementary concept word, anatomy supplementary concept word] | 64,403 |
| 3 | Safety/ or Patient Safety/ or Safety Management/ | 87,165 |
| 4 | Patient Harm/ or Medical Errors/ or Risk Management/ or Medication Errors/ | 49,137 |
| 5 | Quality Indicators, Health Care/ | 17,584 |
| 6 | (patient safety or safety indicator* or quality indicator* or effect or effectiveness or efficiency or patient harm or medication error* or diagnostic error* or medication harm or misdiagnosis or prescription error* or medical error* or safety or quality or comparison or control or management).mp. [mp=title, book title, abstract, original title, name of substance word, subject heading word, floating sub-heading word, keyword heading word, organism supplementary concept word, protocol supplementary concept word, rare disease supplementary concept word, unique identifier, synonyms, population supplementary concept word, anatomy supplementary concept word] | 12,036,356 |
| 7 | Cardiovascular Diseases/ | 183,096 |
| 8 | (hypertension or atherosclerosis or ischemic heart disease or coronary artery disease or angina or dyslipid?emia or hyperlipid?emia or heart failure).mp. [mp=title, book title, abstract, original title, name of substance word, subject heading word, floating sub-heading word, keyword heading word, organism supplementary concept word, protocol supplementary concept word, rare disease supplementary concept word, unique identifier, synonyms, population supplementary concept word, anatomy supplementary concept word] | 1,148,530 |
| 9 | Diabetes Mellitus, Type 1/ or Diabetes Mellitus/ or Diabetes Mellitus, Type 2/ | 387,476 |
| 10 | Hypothyroidism/ or Goiter/ or Hyperthyroidism/ or Thyroid Diseases/ | 75,410 |
| 11 | (hypothyroidism or hyperthyroidism or goiter or diabetes mellitus or diabetes or thyroid disorder*).mp. [mp=title, book title, abstract, original title, name of substance word, subject heading word, floating sub-heading word, keyword heading word, organism supplementary concept word, protocol supplementary concept word, rare disease supplementary concept word, unique identifier, synonyms, population supplementary concept word, anatomy supplementary concept word] | 880,512 |
| 12 | Arthritis, Rheumatoid/ or Arthritis/ or Arthritis, Gouty/ or Arthritis, Psoriatic/ | 148,481 |
| 13 | Osteoarthritis, Hip/ or Osteoarthritis/ or Osteoarthritis, Spine/ or Osteoarthritis, Knee/ | 80,262 |
| 14 | (osteoarthritis or rheumatoid arthritis or gout).mp. [mp=title, book title, abstract, original title, name of substance word, subject heading word, floating sub-heading word, keyword heading word, organism supplementary concept word, protocol supplementary concept word, rare disease supplementary concept word, unique identifier, synonyms, population supplementary concept word, anatomy supplementary concept word] | 252,178 |
| 15 | Neoplasms/ | 517,805 |
| 16 | cancer.mp. [mp=title, book title, abstract, original title, name of substance word, subject heading word, floating sub-heading word, keyword heading word, organism supplementary concept word, protocol supplementary concept word, rare disease supplementary concept word, unique identifier, synonyms, population supplementary concept word, anatomy supplementary concept word] | 2,297,416 |
| 17 | Mental Disorders/ or Depression/ | 331,416 |
| 18 | (mental disorder* or depression or anxiety or bipolar disorder or mood disorder or mental health problem*).mp. [mp=title, book title, abstract, original title, name of substance word, subject heading word, floating sub-heading word, keyword heading word, organism supplementary concept word, protocol supplementary concept word, rare disease supplementary concept word, unique identifier, synonyms, population supplementary concept word, anatomy supplementary concept word] | 923,512 |
| 19 | Lung Diseases/ | 72,459 |
| 20 | (asthma or chronic obstructive pulmonary disease or COPD).mp. [mp=title, book title, abstract, original title, name of substance word, subject heading word, floating sub-heading word, keyword heading word, organism supplementary concept word, protocol supplementary concept word, rare disease supplementary concept word, unique identifier, synonyms, population supplementary concept word, anatomy supplementary concept word] | 273,281 |
| 21 | Kidney Diseases/ or Renal Insufficiency, Chronic/ | 128,121 |
| 22 | (chronic kidney disease* or chronic kidney failure or chronic kidney condition* or renal insufficiency or kidney insufficiency).mp. [mp=title, book title, abstract, original title, name of substance word, subject heading word, floating sub-heading word, keyword heading word, organism supplementary concept word, protocol supplementary concept word, rare disease supplementary concept word, unique identifier, synonyms, population supplementary concept word, anatomy supplementary concept word] | 128,596 |
| 23 | 1 or 2 | 64,403 |
| 24 | 3 or 4 or 5 or 6 | 12,036,356 |
| 25 | 7 or 8 or 9 or 10 or 11 or 12 or 13 or 14 or 15 or 16 or 17 or 18 or 19 or 20 or 21 or 22 | 5,944,378 |
| 26 | 23 and 24 and 25 | 11,041 |
| 27 | Primary Health Care/ | 93,640 |
| 28 | Family Practice/ or General Practice/ | 79,175 |
| 29 | (general practice or primary care or primary health care or family practice or GP).mp. [mp=title, book title, abstract, original title, name of substance word, subject heading word, floating sub-heading word, keyword heading word, organism supplementary concept word, protocol supplementary concept word, rare disease supplementary concept word, unique identifier, synonyms, population supplementary concept word, anatomy supplementary concept word] | 323,988 |
| 30 | 27 or 28 or 29 | 323,988 |
| 31 | 26 and 30 | 1,222 |
| 32 | limit 31 to yr="2014 - 2024" | 957, after duplicate removal 955 |
